# Understanding Host–Pathogen Interactions in *Brassica napus* in the Omics Era

**DOI:** 10.3390/plants9101336

**Published:** 2020-10-10

**Authors:** Ting Xiang Neik, Junrey Amas, Martin Barbetti, David Edwards, Jacqueline Batley

**Affiliations:** 1 Sunway College Kuala Lumpur, Bandar Sunway 47500, Selangor, Malaysia; tingxiang@gmail.com; 2 School of Biological Sciences and Institute of Agriculture, The University of Western Australia, Perth 6009, Australia; junrey.amas@research.uwa.edu.au (J.A.); dave.edwards@uwa.edu.au (D.E.); 3 School of Agriculture and Environment and Institute of Agriculture, The University of Western Australia, Perth 6009, Australia; martin.barbetti@uwa.edu.au

**Keywords:** *Brassica napus*, host–pathogen interaction, pathosystems, omics, next-generation sequencing (NGS), pangenomics, secretomics, bioinformatics

## Abstract

*Brassica napus* (canola/oilseed rape/rapeseed) is an economically important crop, mostly found in temperate and sub-tropical regions, that is cultivated widely for its edible oil. Major diseases of *Brassica* crops such as Blackleg, Clubroot, Sclerotinia Stem Rot, Downy Mildew, Alternaria Leaf Spot and White Rust have caused significant yield and economic losses in rapeseed-producing countries worldwide, exacerbated by global climate change, and, if not remedied effectively, will threaten global food security. To gain further insights into the host–pathogen interactions in relation to *Brassica* diseases, it is critical that we review current knowledge in this area and discuss how omics technologies can offer promising results and help to push boundaries in our understanding of the resistance mechanisms. Omics technologies, such as genomics, proteomics, transcriptomics and metabolomics approaches, allow us to understand the host and pathogen, as well as the interaction between the two species at a deeper level. With these integrated data in multi-omics and systems biology, we are able to breed high-quality disease-resistant *Brassica* crops in a more holistic, targeted and accurate way.

## 1. Introduction

Plants interact very closely with microorganisms, such as fungi and bacteria, in the natural environment through a symbiotic relationship with endophytes, often having beneficial effects on the plant [1]. By contrast, a parasitic relationship with pathogenic microbes has harmful effects on the plant. In a parasitic relationship, the plant plays host while the fungal or bacterial pathogen feeds off the nutrients from the host at the host’s expense. Depending on the mode of nutrition, plant pathogens are classified as (a) biotrophic, where the pathogen obtains nutrients from the living plant without causing the plant to die; (b) necrotrophic, where the pathogen kills off the plant and utilizes the nutrients that are released; or (c) hemibiotrophic, where the pathogen transitions from biotrophic to necrotrophic in different stages of invasion in the host [2].

Plants, in response, protect themselves from pathogen attack typically through two divisions of immunity. The first is the innate immune response, also known as PAMP (pathogen-associated molecular pattern)-triggered immunity (PTI). This provides defence against a broad range of pathogens involving cell surface plant pattern recognition receptors (PRRs) recognising extracellular PAMPs. The second is adaptive immunity, also referred to as effector-triggered immunity (ETI). This provides complete resistance to the host and is mediated by a specific interaction between the resistance (*R*) gene in the host and the effector (Avirulence, *Avr*) gene in the pathogen [3,4]. The tight relationship between the host and the pathogen is a result of a long-term co-evolutionary process where the fungal pathogen and the host plant each strive to keep ahead by evolving ways to overcome resistance/pathogenicity [5], as described in the PTI/ETI zigzag model of the plant immune system [6]. Newer models, such as “effector-triggered defence” (ETD) [7], the “invasion model” [8], the “spatial immunity model” [9] and the “spatial invasion model” [10], have also been proposed. These accommodate the recognition of pathogens at the host immune receptors at a wider level, not restricted to the assignment of the PTI response to PRR proteins and the ETI response to *R* genes, during defence signalling.

*Brassica napus* is an allopolyploid (AACC genome, *n* = 19) belonging to the family Brassicaceae. The Brassicaceae constitute 325 genera and 3740 species [11,12], with the genus *Brassica* having 73 accepted species [13,14]. The six most important *Brassica* species are *B. rapa* (AA genome), *B. nigra* (BB genome), *B. oleracea* (CC genome), *B. napus* (AACC genome), *B. juncea* (AABB genome) and *B. carinata* (BBCC genome). These six species are cultivated as vegetables, oilseeds and condiments, and display a wide variety of morphotypes, including as leafy vegetables such as Chinese cabbage and pak choi in *B. rapa*, the enlarged inflorescences of cauliflower and broccoli in *B. oleracea,* and the condiment seeds of mustard plants in *B. nigra* and *B. juncea* [15,16]. *B. napus* is an oilseed crop that is traded globally and a major cash crop [14]. *B. napus* is widely grown in Europe, Canada, China and Australia [17] and ranks second after the soybean in terms of world oilseed production (75,001,457 tonnes vs. 348,712,311 tonnes) [18]. There is a pressing need for improving *B. napus* crop yield for high-quality and sustainable production to meet the growing demand of food consumption. This faces two challenges—first, the predicted increase in the human population to 9 billion by 2050, and second, the impact of unpredicted weather patterns worldwide due to global climate change [19,20].

**Figure 1:**
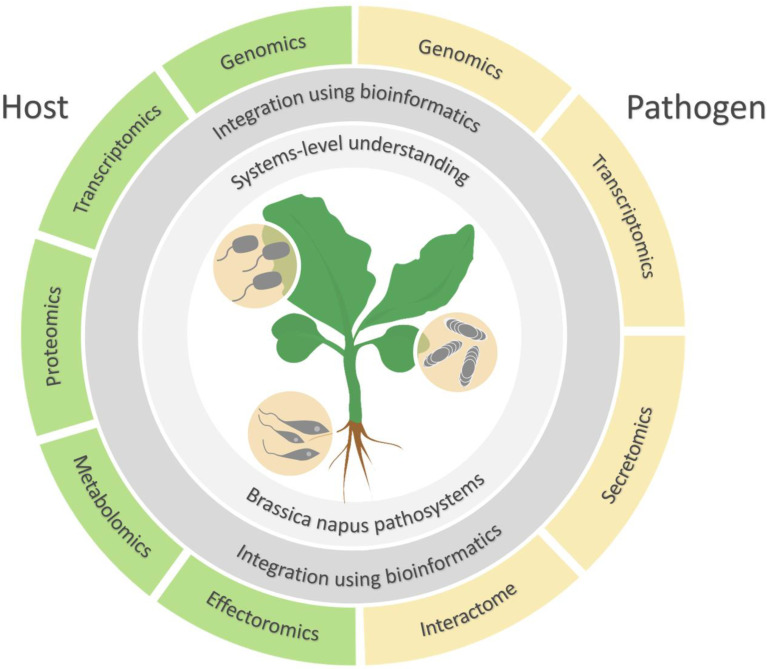
The integration of omics studies in *B. napus* pathosystems.

*B. napus* plays host to several fungal pathogens that cause major diseases leading to substantial loss in global production. These diseases include Blackleg (hemibiotrophic fungal pathogen *Leptosphaeria maculans*), Clubroot (obligate biotrophic protist/chytrid *Plasmodiophora brassicae*), Sclerotinia Stem Rot (necrotrophic and more recently proposed as a hemibiotrophic fungal pathogen *Sclerotinia sclerotiorum*) [21], Downy Mildew (obligate biotrophic oomycete *Hyaloperonospora brassicae*), Alternaria Leaf Spot (necrotrophic fungal pathogen, particularly *A. brassicae* but also *A. alternata* and a range of other *Alternaria* spp.), and White Rust (obligate biotrophic oomycete *Albugo candida*) [22,23]. These diseases have widely caused a yield loss of 24–50% and economic loss of up to USD 200 million in the *B. napus* industry, with the potential to wipe out the entire crop where not effectively controlled [24,25,26,27,28,29]. The most promising approach to controlling diseases of *Brassica* is through breeding disease-resistant varieties [30]. In this respect, the omics studies of *B. napus* pathosystems, integrating technologies from both the host and pathogen, are pertinent in breeding *Brassica* varieties that are better able to resist pathogen attack (Figure 1).

Here, we review the current status of the application of omics technologies to understand the molecular aspects of host–pathogen interactions in *B. napus* and other *Brassica* crops. We first discuss the application of omics technologies in the host, in relation to the identification of candidate QTL/*R* genes. We then discuss progress in pathogen research, focusing on the application of omics tools in the discovery of pathogenicity genes. Lastly, we review future perspectives and prospects for the utilisation of omics technologies in *Brassica*–pathogen research for breeding high-quality, more-resistant crop varieties.

## 2. Application of Omics Technologies in *Brassica* Host Plants

### 2.1. High-Quality Genome Assemblies

With the introduction of next-generation sequencing (NGS) technology, five out of six *Brassica* crop genomes (*B. rapa*, *B. oleracea*, *B. nigra*, *B. napus* and *B. juncea*) have now been sequenced, with some species having more than one genome from different individuals [31,32,33]. The field of *Brassica* genomics has been “revolutionised” by the development of long-read sequencing technologies such as PacBio Single Molecule, Real-Time (PacBio) sequencing [34] and Oxford Nanopore Technologies (ONT) [35], along with high-throughput physical mapping technologies such as BioNano optical mapping [36] and Chromosome Conformation Capture (Hi-C) [37,38]. Valuable genomics resources for interrogating the molecular aspects of *Brassica*–pathogen interactions have been provided via high-quality genome assemblies of *Brassica* species using PacBio sequencing, for example, *B. rapa* cultivar “Chiifu-401-42” [39], *B. oleracea* cultivar “C-8” [40], *B. napus* German winter cultivar “Express 617” [33] and the recent improved *B. napus* “Darmor-*bzh*” [41], along with the highly contiguous *B. nigra* assembly, both achieved via ONT technology (Table 1). These high-quality *Brassica* assemblies resolve the “difficult” genomic regions commonly found in polyploid crops [42], particularly highly repetitive DNA sequences related to transposable elements (TEs), copy number variation (CNV), presence–absence variation (PAV) and homoeologous exchange, many of which are associated with disease resistance genes [43,44,45].

Although high-quality *Brassica* genome assemblies are currently available, such reference genomes and other *Brassica* genomes that were previously sequenced represent only a fraction of the *Brassica* morphotypes. For example, the reference *B. rapa* “Chiifu” is a heading type [39], while the reference “Z1” is a sarson type [46], the cauliflower *B. oleracea* “C-8” is an inflorescence type [40], and “TO1000” is a leafy type [47]. That genomes have not yet been assembled for other morphotypes in *Brassica* species, such as root or stem tubers in *B. rapa* (turnip), *B. oleracea* (kohlrabi) and *B. napus* (swede) [16], means that we may be missing out on much of the extensive genetic diversity present within the various *Brassica* species, but also that a wealth of novel alleles for disease resistance are potentially overlooked if we rely on a single reference genome. These issues have driven the development of pangenomes in plants.

### 2.2. Pangenomics

Pangenomics has been developed to overcome the limitations of relying on a single reference genome, and allows more comprehensive genomic variations to be identified from the gene pools represented by many lines within a species [48,49,50]. The recent pangenome of *B. napus,* which was built with eight *B. napus* lines encompassing three ecotypes using a de novo approach, showed the PAV regions were enriched with genes associated with a defence-related response [51]. Furthermore, in the *B. oleracea* and *B. napus* pangenomes, it was found that a large proportion of the disease resistance genes were dispensable, meaning that these genes are not present in all lines [44,52] (Table 1). These findings suggest that the *R* genes in *Brassica* are highly variable, resulting from the strong selection pressure of arms-race evolution during host–pathogen interaction, superimposed by the frequent homoeologous exchanges between sub-genomes [44] during the domestication process within the *Brassica* lineage [15,50]. Hence, many candidate *R* genes may have been missed from a single reference genome, thus hindering the speed of *R* gene cloning in *Brassica* crops.

Pangenomics has identified a large *R* gene repertoire, collectively known as resistance gene analogues (RGAs), in *Brassica* species. Examples of RGAs include nucleotide-binding site leucine-rich repeats (NLRs), mainly comprising the TIR-NBS-LRR (TNL) and CC-NBS-LRR (CNL) types, receptor-like protein kinases (RLKs), receptor-like proteins (RLPs) and wall-associated kinases [53,54]. Using pangenomics, 106 RGAs have been identified within the Blackleg QTL in the *B. napus* pangenome [45] while 59 RGAs were detected within the Sclerotinia, Fusarium wilt and Clubroot resistance QTLs in the *B. oleracea* pangenome [43]. These pangenomics studies revealed that different classes of RGAs (RLKs, TNLs and others) show different percentages of variability across the lines, which leads to the following question: is there any association between *R* gene variability and resistance outcomes in *Brassica* crops for the major diseases? It was also found in the *B. oleracea* pangenome study that the wild relative *B. macrocarpa* harbours the most RGA candidates, suggesting that a large pool of genetic resources for *R* genes can be found in wild *Brassicas* [43,52]. Recently, a super-pangenome was reported that includes the genomes of wild relatives and/or different species within a genus, which adds an additional level of depth for investigating genomic variations within a crop genus [55]. With a super-pangenome developed in *Brassica* crops, not only can we identify many more novel candidate disease resistance genes from the wild genotypes, but we can also develop molecular markers to screen for resistant varieties in the field, therefore not only improving the speed and accuracy of crop breeding but also broadening the *Brassica* gene pool by using novel alleles from wild germplasm.

**Table 1:** Summary of the most recent *Brassica* reference genomes useful for omics studies in the *Brassica* pathosystems.

Reference Genome	Approach	Major Findings Relevant to *R* Gene Study	Reference
**Single genome**			
*B. napus* winter cultivar “Express 617”	PacBio, ONT, Illumina HiSeq, Optical mapping	Resolved break-point sequence at homoeologous exchange regions	Lee, et al. [33]
*B. oleracea* cultivar “C-8”	PacBio, Illumina HiSeq, transcriptomics	Cauliflower is the most recent var. to evolve within *Brassica* genus. It contains more repetitive sequences compared to other *B. oleracea* species	Sun, et al. [40]
*B. nigra* accession CGN7651	ONT, Hi-C	Hotspot of ALE-type retroelement in the centromeric regions showed that these retroelements play an important role in the divergence of *B. nigra* centromere	Perumal, et al. [56]
*B. rapa* cultivar “Chiifu-401-42”	PacBio, Optical mapping, Hi-C	V3.0, improved repeat reads, defined locations of centromeres and annotated more genes in these difficult regions. Annotated higher number of TEs	Zhang, et al. [39]
**Pangenome**			
Eight *B. napus* accessions of three ecotypes	Alignment of de novo assembled genomes against “ZS11”	PAV genes were highly represented by defence response gene	Song, et al. [51]
33 non-synthetic and 20 synthetic *B. napus* accessions	Iterative mapping and assembly using improved “Darmor-bzh”(v8.1) from Bayer, Hurgobin, Golicz, Chan, Yuan, Lee, Renton, Meng, Li, Long, Zou, Bancroft, Chalhoub, King, Batley and Edwards [31] as reference	Homoeologous exchange-related PAV genes highly represented by defence, stress and auxin pathways	Hurgobin, et al. [44]
Nine *B. oleracea* subspecies and wild type comprising cabbage, kale, Brussels sprouts, kohlrabi, cauliflower, broccoli and *B. macrocarpa*	Iterative mapping and assembly using Chinese kale rapid cycling line (TO1000) as reference	18.7% of genes showed PAV with annotation of disease resistance genes	Golicz, et al. [52]
Two *B. rapa* subspecies: turnip and rapid cycling	Alignment of de novo assembled genomes against “Chiifu” reference	Peroxidase genes that are involved in phenylpropanoid biosynthesis response pathway during biotic stress are unique in turnip, with evidence of copy number variation	Lin, et al. [57]

### 2.3. Identification of Candidate QTLs/Genes Using NGS-Based SNP Methods

In the past two decades, many genetic linkage maps of *Brassica* crops have been generated, using bi-parental crossing, selfing and backcrosses, with molecular markers such as Restriction Fragment Length Polymorphisms (RFLPs), Amplified Fragment Length Polymorphisms (AFLPs) and Randomly Amplified Polymorphic DNA (RAPDs) reviewed by Delourme, et al. [58]. These molecular markers are often limited by low reproducibility and laborious techniques, thus limiting the quality of marker information in *Brassica* crops [59]. The genomics era, driven by the innovation of high-throughput next-generation sequencing (NGS) technologies, has significantly increased the efficiency of the identification of QTL/candidate genes for disease resistance in *Brassica* crops through the development of genome-wide DNA-based molecular markers. This has brought great improvement in the resolution of genetic maps [60].

Among the DNA-based molecular markers, single nucleotide polymorphism (SNP) markers are most widely used for determining genotypic variation in a given species because they are uniformly distributed and highly abundant in the genome and are amenable in multiple genotyping platforms [61,62]. With NGS, high-throughput genome-wide SNP marker development can be achieved rapidly and accurately in *Brassica* species through systems such as whole-genome resequencing (WGRS), genotyping-by-sequencing (GBS), and the Brassica 60K Illumina Infinium^TM^ 60K SNP array [63].

WGRS is an omics strategy for obtaining high-quality, high-density SNP markers at a whole-genome level by mapping sequence reads to the *Brassica* reference genome assemblies [64]. In GBS, restriction enzymes are used to digest the genomic DNA and barcodes are used to ligate the fragmented DNA molecules before whole-genome sequencing is performed for SNP discovery [65]. In this way, GBS is less complicated compared to WGRS because the sequencing reads cover only part of the genome instead of the whole genome, thus offering a cost-efficient approach to identifying SNPs yet achieving equally high-quality SNPs with wide applications in crop improvement studies [63,66]. An extension of GBS called tGBS^®^ using oligonucleotides instead of adaptors has since been developed [67]. The Brassica 60K SNP array offers a whole-genome SNP genotyping approach that is highly reproducible for genotyping hundreds of DNA samples in 48 h, making it an attractive option for the routine screening of *Brassica* germplasm [68,69,70]. A Brassica 60K SNP array data repository called CropSNPdb has been developed to enable users to access SNP information, currently containing genotypic information of 526 *Brassica* lines [71], permitting the easy retrieval of whole-genome SNP data for a wide range of downstream data analyses.

These NGS-based SNP genotyping approaches have been applied widely for the QTL mapping of disease resistance traits and identification of candidate genes through genome-wide association studies (GWAS) in *Brassica* crops (Table 2). Highlights include the discovery of novel disease resistance QTLs/genes at an unprecedented speed, for example, in Blackleg [72,73], Sclerotinia [74,75,76] and Clubroot [77,78,79]. The other benefit of the application of NGS-based SNP genotyping is the successful breeding of *B. napus* varieties containing multiple improved traits. For example, breeding was achieved through the introgression of several major QTLs for Sclerotinia quantitative resistance from *B. oleracea* into *B. napus* with good seed yield and quality [80]. In addition, bioinformatics pipelines are continuously being improved for whole-genome SNP data analysis. One example is Single Nucleotide Absence Polymorphism (SNaP) analysis, which successfully recovered numerous QTLs for Sclerotinia and Blackleg resistance in *B. napus* that were lost from the normal filtering of SNP data obtained from the Brassica 60K SNP array, with 3.2- and 2.2-fold increases in significant marker-trait associations for Sclerotinia and Blackleg resistance, respectively [81].

### 2.4. Identification of Candidate R Gene Using In Silico Methods

The large volume of *Brassica* genomics resources in public databases supports the analysis and interpretation of many complex mechanisms related to *Brassica*–pathogen interaction. Examples include the in silico identification of the Blackleg resistance gene, *LepR4*, in the C genome of Korean cabbage (*B. oleracea* var. *capitata*) [88] and the in silico exploration of 641 NBS-LRR-type disease resistance genes in *B. napus*, together highlighting the genomic distribution and structural variation of these genes in *B. napus* [89]. Other examples include the in silico evolutionary study of NBS genes in *B. napus*, where comparative genomic analysis highlighted the NBS gene’s distribution from its progenitors *B. rapa* and *B. oleracea* in relation to the three main *Brassica* diseases—Blackleg, Clubroot and Sclerotinia [90]. Coupled with modern bioinformatics tools and the integration of multi-omics data sets, in silico methods are powerful tools that rapidly provide accurate and detailed models to answer various research questions ranging from candidate gene identification to evolutionary pathways of resistance mechanisms in both the *Brassica* host and the fungal pathogens [91]. This is a time-, cost- and manpower-effective means of conducting higher-quality *Brassica* crop improvement research investigations.

In addition, database searches for protein motifs associated with disease resistance genes have enabled researchers to identify classes of *R* genes in *B. napus* that are associated with Blackleg, Sclerotinia and Clubroot resistance [92]. Stotz, et al. [92] suggest most Clubroot resistance genes are NLR type; Blackleg resistance genes are RLP type, while for Sclerotinia, neither NLR nor RLP were involved. Focusing on the NLR genes, comparative genomics and transcriptomics analyses supplemented with a database query on *B. napus* and its progenitors, *B. rapa* and *B. oleracea,* revealed many more NBS (also known as NLR) genes in the C sub-genome of *B. napus.* A number of these genes underly the QTL regions for resistance against Blackleg, Sclerotinia and Clubroot, supporting the concept that the diversification of the *R* genes likely happened after interspecific hybridisation between *B. rapa* and *B. oleracea* [90].

### 2.5. NGS-Based Bulked Segregant Analysis (BSA)

NGS-based BSA is one of the more recent applications of omics in studying *Brassica*–pathogen interactions. This technique involves bulks/pools of DNA samples with representations of individuals with segregating phenotypes, where the pools are genotyped using NGS, either RNA sequencing (BSR-Seq) or whole-genome resequencing, followed by the detection of QTLs through SNP calling between the bulks (QTL-Seq) [93,94]. The traditional BSA technique allows the screening of many loci. An example is screening for Downy Mildew resistance in lettuce [95], but it is restricted to the detection of random sequence variation (e.g., RFLPs) and requires intense PCR screening efforts to confirm the molecular markers that are linked with the selected genomic intervals. With the NGS screening of BSA populations, the detection of sequence polymorphisms between the bulks is rapid and effective, as novel variation, such as PAVs or even novel QTLs/genes, can potentially be detected [96].

Using BSR-Seq, an *R* gene for resistance against Blackleg, *Rlm1,* was fine mapped in the *B. napus* cultivar “Quinta”, and a candidate gene was identified, BnA07G27460D, that encodes a serine/threonine protein kinase. This gene is homologous to the protein kinase STN7 in *B. rapa*, *B. oleracea* and *A. thaliana*, which is involved in systemic plant immune responses by regulating reactive oxygen species (ROS)-induced cell signalling at the thylakoid membrane [97]. BSR-Seq has also been applied in characterising Clubroot resistance in some *Brassica* speciesl; for example, in *B. oleracea*, the first Clubroot major *R* gene (*Rcr7*) in the *B. oleracea* cultivar “Tekila” was identified; Bo7g108760 was the candidate TNL gene [98]. In Chinese cabbage *B. rapa* var. *pekinensis*, the candidate gene *Rcr2,* which encodes a TIR-NBS-LRR, responsible for Clubroot resistance, has been identified on chromosome A03 [99]. In *B. nigra*, a novel Clubroot *R* gene, *Rcr6*, was detected (BniBo15819, encoding a TNL gene), which is homologous to chromosome A08 of *B. rapa* and which provides a good source for gene introgression into *B. napus* [100]. Using QTL-Seq, two novel QTL regions associated with Clubroot resistance on chromosomes A07 and A08 were detected in pak choi *B. campestris* var. *chinensis* [96]. Lastly, a single novel candidate *R* gene, also involved in Clubroot resistance, *CRd,* was identified on chromosome A03 of *B. rapa* [101].

### 2.6. Resistance Gene Enrichment and Sequencing (RenSeq)

RenSeq is a targeted resequencing method for identifying NLRs [102]. RenSeq in combination with PacBio sequencing was applied in *A. thaliana* to study the *R* gene sequence variants of the White Rust Resistance (WRR) gene against *Albugo candida* [103]. This combinatorial approach was extended to 64 accessions of *A. thaliana* to study the evolution and variability of NLR genes in the model plant *Arabidopsis*, resulting in the construction of a species-wide pan-NLR-ome [104]. Similar to the concept of pangenomics, the pan-NLR-ome is the collection of all the NLR genes and alleles contained within a species, distinguishing between the core and non-core NLRs in terms of the structural variations and diversity. New domain structures of NLRs were identified from the pan-NLR-ome study in *A. thaliana* [104]. This implies that novel *R* genes can be obtained from the diverse gene pool of NLRs within members of the Brassicaceae family, including *Brassica* crops. The discovery of a repertoire of NLRs is particularly important when pathogen-specific recognition can not only happen in the host species but also in the non-host species. For example, the non-host *A. thaliana* displayed ETI-mediated defence against *A. candida* isolates derived from *B. juncea*, *B. rapa* and *B. oleracea* [103], implying that *R* genes play a conserved role across members within the same family. This implies that screening for novel *R* genes for *Brassica* crop improvement should also be applied across non-host species. Pan-NLR-ome type studies contribute significantly towards the discovery of *R* gene diversity in crops.

### 2.7. Effectoromics

Effectoromics, or effector-based screening, is an omics approach to detecting *R* genes in crop plants, although *R* gene products may not necessarily interact with effectors directly. In this method, the target effector, as forecast from prediction tools, is transformed using *Agrobacterium* and infiltrated onto the host plants. The host genotypes that give a positive response to the target effector are then subjected to resistance gene mapping using molecular markers [105]. Effectoromics allows screening for potential recognition targets in a particular crop species, for example, immune receptors, and can be applied to screen different crop species with a selection of potential candidates that can be used in inter-crop species [106]. This method was used for the identification of *R* genes in the wild potato species *S. pinnatisectum* against the oomycete *Phytophthora infestans* [107,108] and could be applied to *Brassica* pathosystems for the identification of *R* genes.

### 2.8. Transcriptomics

Plant–pathogen interactions are complex, involving a breadth of interconnected molecular mechanisms [109]. Critical for understanding these mechanisms is deciphering which genes are activated in both the host and the pathogen during infection and how these genes affect the expression of others in the pathways. Transcriptome analysis has allowed the monitoring of the molecular cues involved in these interactions.

Gene expression analysis has already significantly evolved since early techniques were described and developed [110]. Recent approaches, including RNA sequencing or RNA-seq, overcome the limitations of previous techniques and are considered superior to predecessors in that they can interrogate the whole genome transcriptome of any organism with or without reference genomes and facilitate the discovery of unique genes [111]. They are highly sensitive in detecting lowly-expressed genes and have been shown to be highly reproducible. These features make RNA-seq the method of choice in most transcriptome studies, including the analysis of host–pathogen interactions. An advance in this approach, called dual-RNA seq, enables the simultaneous study of gene expression in both the host and pathogen [112,113], enabling a real-time and comprehensive analysis of the mechanisms involved in both pathogenesis and the host resistance response.

Furthermore, integrative approaches such as associative transcriptomics (AT) [114] and bulked RNA sequencing (BSR-Seq) [97] have allowed the incorporation of transcriptome data with genome-wide marker information to increase the power of detection for genomic loci controlling resistance or susceptibility in the host and virulence in the pathogen. These approaches have facilitated a fast-tracked identification of candidate genes underlying these loci and greatly facilitated the dissection of gene expression patterns during pathogen attack in important *Brassica* crops. AT is an RNA-based approach that integrates transcriptome data in GWAS to identify molecular markers associated with a particular trait of interest at marker loci where the levels of gene sequence and gene expression are variable [114]. AT is particularly useful for dissecting trait variation in polyploid species characterized as containing highly duplicated genes displaying variable expression patterns [115].

In *B. napus* resistance against Blackleg, three of the known *R* genes have been cloned and were found to encode Leucine-Rich Repeats-Receptor Like Proteins (LRR-RLPs; *Rlm2* and *LepR3*) and a wall-associated kinase-like (WAKL) protein (*Rlm9*) [116,117]. The cloning of these genes provided the starting material for using RNA-seq to interrogate the detailed machinery involved in the resistance. The global transcriptome analysis of Zhou, et al. [118] found that both *LepR3* and *Rlm2* evoked a basal defence response in both compatible and incompatible interactions upon inoculation with *L. maculans* isolates. This suggests that *LepR3* and *Rlm2* may also monitor other molecular patterns produced by *L. maculans* to mount a resistance response in the host plant.

The *Rlm9* WAKL protein is a type of receptor-like kinase (RLK) localized in the cell wall, which functions to sense environmental and cellular signals [119]. *Rlm9* is only the second WAKL *R*-gene identified to date; hence, the mechanisms underlying its resistance are yet to be studied in detail. Initially, Larkan, et al. [117] found *Rlm9* did not seem to have a direct interaction with its counterpart *Avr* gene (*AvrLm5-9*), and it is likely that a mediator molecule, such as damage-associated molecular patterns (DAMPs), is needed to effect resistance. This is supported by the findings of Brutus, et al. [120], which showed that WAKLs can detect DAMPs following pathogen attack. However, this latter mechanism needs to be verified in further studies. Genome-wide transcriptome analysis for this interaction should help uncover how *Rlm9* orchestrates race-specific resistance responses against the Blackleg pathogen. Furthermore, as *Rlm9* forms part of the tightly linked *R*-gene cluster (*Rlm3*/*4*/*7*/*9*) on chromosome A07 of *B. napus*, the cloning of this gene may enable an understanding of the molecular mechanisms of the other genes on this cluster.

The introgression of the major genes *Rlm2*, *Rlm3*, *LepR1* and *LepR2* in cultivar “Topas” and *LepR1* and *LepR2* in “Westar” allowed the comparison of the effect of genetic background and *R* gene content in host defence expression through genome-wide transcriptome profiling by Haddadi, et al. [121]. All the introgression lines (ILs) showed an upregulation of genes previously implicated in host defence, including hormone signalling, cell wall thickening, chitin production, and glucosinolate production. Interestingly, these genes have higher levels of expression in *LepR1* and *Rlm2* compared with *LepR2* and *Rlm3* lines during the first three days of infection. Furthermore, a general trend of delayed defence responses in “Westar” compared with “Topas” ILs was observed, regardless of their *R* gene content. This suggests that the genetic background has important effects on resistance. Additionally, there was enhanced expression of the RLK *Brassica napus* (Bn) SOBIR1 (Suppressor of BIR1-1) and salicylic acid-related defence in both *LepR1* and *Rlm2* lines, consistent with previous investigations [116,118,122]. Transcriptomic studies also showed the involvement of host receptor genes, for example, RLPs, RLKs, TIR-NBS and WAKLs, in PTI or effector-triggered defence (ETD) in the *B. napus–L. maculans* interaction [123]. All these studies suggest that *Avr* genes in *L. maculans* likely play a role in manipulating host resistance in the apoplast environment. The AvrLm1 protein reportedly interacts with the mitogen-activated protein kinase (MAPK) 9 in *B. napus* (BnMPK9) [124]. Furthermore, MAPK9 is implicated as a positive regulator of ROS-mediated abscisic acid (ABA) signalling in the guard cells of the plant, fostering stomatal closure [125,126]. Larkan, Ma and Borhan [116] previously reported the association of *Bn-SOBIR* with *Rlm2*, and it was assumed that the *Rlm2* and *Bn-SOBIR* interaction results in downstream signalling to effect race-specific resistance against the *AvrLm2 L. maculans* pathotype. Given this information, it is likely that *LepR1* conveys resistance through the same mechanism. Conversely, the expression of *Bn-SOBIR* was low in *Rlm3* introgression lines, which means that *Rlm3* not only functions independently of the SOBIR1 interaction but represents another resistance mechanism different from that of other cloned genes. The cloning and transcriptomic analysis of this gene will shed light on the molecular mechanisms governing the operation of this resistance [121].

In the *B. napus*–Clubroot pathosystem, several *R* genes acting against *P. brassicae* have been mapped, but only two have been cloned: *CRa* [127] and *Crr1a* [128], which encode TIR-NBS-LRRs. Plant hormones such as ethylene (ET), jasmonic acid (JA), salicylic acid (SA), abscisic acid (ABA), auxin and cytokinin were implicated in the pyramided lines of *B. napus* containing two Clubroot resistance genes, *PbBa8.1* and *CRb,* with the candidate genes involved in the hormone signalling pathway identified through comparative RNA-seq [129]. The transcriptome analysis in *CRb*-containing *B. rapa* lines, at the early stages of *P. brassicae* infection, confirmed the involvement of pathways typical for ETI-mediated resistance and biotrophic infection [130]. These include the activation of NLR and pathogenesis-related (PR) genes, along with the upregulation of genes for MAPK, WRKY transcription factors, calcium-binding proteins, chitinases, and SA pathway genes [131,132,133]. However, the transcriptome analysis of Chu, et al. [134] highlighted the induction of JA and ET pathways, implicated in the necrotrophic stage of infection, as important mechanisms of *Rcr1*-mediated resistance in *B. rapa*, thus highlighting a complex molecular mechanism for *P. brassicae* resistance in *Brassica* crops. The cloning of the *Rcr1* gene will help to elucidate these hormones’ induction dynamics. However, whilst *Crr1a* and *CRa* have been cloned, the transcriptional control of their resistance has not been widely researched. Genome-wide transcriptome studies should help to elucidate the resistance mechanisms involved in these two key clubroot *R* genes. To study the genetic effects of the multiple *R* genes in pyramided lines and confirm the role of these introgressed genes in host resistance responses, comparative RNA sequencing could be performed. For example, when two Clubroot-resistant genes, *PbBa8.1* and *CRb,* were introgressed into a *B. napus*-pyramided line, through comparative RNA sequencing, it was found not only that the pyramided lines displayed a strong multi-gene resistance network during pathogen infection, but also that SA- and ROS-mediated resistance responses played a dominant role in the pyramided lines [129], supported by Galindo-González, et al. [135]’s study, highlighting the importance of SA-mediated resistance in the *B. napus*–*P. brassicae* pathosystem.

In a study of Sclerotinia resistance in *B. napus*, Qasim, et al. [136] detected at least 36 candidate genes representing diverse molecular functions in the resistance response, including TIR-NBS-LRR genes, hormone synthesis, the production of secondary metabolites, and the regulation of transcription factors and several metabolic pathways. One metabolic gene involved in the regulation of the phenylpropanoid pathway that plays a key role in lignin biosynthesis was highly transcribed across time points, and one TIR-NBS-LRR gene, in one of the QTL regions, a widely known *R* gene that is associated with a qualitative response, was highly transcribed [136]. As Sclerotinia resistance has been known to be quantitatively controlled, the diversity of *R*-gene host-mediated resistance mechanisms shown in the study mirrors the complexity of quantitative resistance. Some of the described mechanisms are atypical of PTI- and ETI-mediated host defences, supporting an ongoing discussion challenging the applicability of the conventional two-tier model of plant immunity in explaining quantitative resistance variation. This enigma may be due to the differences in the hosts and the pathogens, as well as the approaches employed for studying genome-wide gene expression. Nevertheless, the increasing availability of transcriptome data generated through various high-throughput platforms results in a better comprehension of the mechanisms underpinning quantitative resistance. Global transcription sequencing also revealed that JA and ET signalling were associated with a resistance response against *S. sclerotiorum* in *B. napus* [137,138]; a further transcriptome study in the same pathosystem demonstrated the downregulation of *B. napus* NPR1-like gene, *BnaNPR1*, which plays a role in SA and JA signalling, indicating that *S. sclerotiorum* suppresses the expression of *BnaNPR1* during systemic acquired resistance (SAR) for successful invasion into the host cell [139]. It is also noteworthy that NPR1 genes were activated by NBS-LRR genes, as reported in a gene pyramiding study in *B. napus* using two NBS-LRR genes, *BvHs1^pro−1^* and *BvcZR3*, obtained from nematode (*Heterodera schachtii*)-resistant sugar beet [140]. The gene interaction network of NPR1 and NBS-LRR should be studied further in relation to other defence-related genes.

Comparative transcriptomic analysis of the *B. napus*–*S. sclerotiorum* pathosystem showed that indolic glucosinolate biosynthesis plays an important role [137], similar to that found from an overexpression experiment with three glucosinolate genes in *B. napus*, one of which, *BnUGT74B1*, encoding cytochrome P450, enhanced resistance to *S. sclerotiorum* [141]. Transcriptomic analysis in the *B. oleracea*–*S. sclerotiorum* interaction revealed a total of 45 *B. oleracea* genes involved in Ca^2+^ signalling were upregulated, which is important in ROS generation [142], and this is consistent with the findings that the resistance mechanism in *B. oleracea* showed ROS generation and increased Ca^2+^ signalling contributing towards resistance outcomes in *B. oleracea* [143].

A transcriptome study of Downy Mildew (*H. brassicae*)-infected Chinese cabbage lines demonstrated the involvement of protein processing in endoplasmic reticulum and circadian rhythm pathways in resistance mechanisms [144]. In addition, the authors found the downregulation of photosynthetic genes during *H. brassicae* infection, which is consistent with Xiao, et al. [145], who reported a downregulation of energy metabolism genes, particularly those involved in the photosynthetic carbon cycle (PCC). This indicates that the resistance to *H. brassicae* in *Brassica* crops may be driven by efficient energy metabolism during pathogen invasion.

In an RNA-seq study of *B. oleracea*–*Fusarium oxysporum* f. sp. *Conglutinans* interactions, Ca^2+^-binding ATPase and aquaporin tonoplast intrinsic protein (TIP), which are involved in Ca^2+^ signalling, were highly expressed in the resistant genotype at 4 h after infection [146]. Similarly, the experiment of Tortosa, et al. [147], which examined the transcriptome dynamics in *B. oleracea* in response to the black rot pathogen (*X. campestris*), highlighted the role of Ca^2+^ signalling proteins as secondary messengers for several downstream signalling processes, including the activation of several transcription factors responsible for the initiation of SA-mediated host defence. The deep RNA-seq of Liu, et al. [148] found an upregulation of several plant pathogen receptor genes such as chitin elicitor receptor kinase 1, chitin receptor, LRR-RLPK and WAKL, which are important in the PTI defence response. This led them to conclude that PTI is the primary mechanism for soft rot resistance in Chinese cabbage (*B. rapa* var. *pekinensis*), initiating several downstream signalling pathways for hormone regulation and the production of secondary metabolites and cell wall reinforcement.

### 2.9. Proteomics

The use of 2D gel electrophoresis (2-DGE) and matrix-assisted laser desorption/ionisation time-of-flight mass spectrometry (MALDI-TOF/TOF MS) technology to study the proteomics of plant–pathogen interactions was first reported more than a decade ago [149]. Comparative proteomic analysis of responses to *L. maculans,* between compatible and incompatible interactions in *B. napus* cv. “Surpass 400” with either the virulent isolate UWA 192 or avirulent isolate UWAP11, showed the upregulation of enzymes involved in RuBisCO for CO_2_ fixation, H_2_O_2_ scavenging and redox metabolism [150]. In *L. maculans*-tolerant *B. carinata*, most of the proteins displayed antioxidant activities [151]. Similarly, in the *B. carinata–L. maculans* interaction, it was found from 2-DGE analysis that proteins related to ROS generation and photosynthetic enzymes were elevated in the resistant genotype 48 h after pathogen infection [152]. A proteomics study in the *B. rapa*–*P. brassicae* pathosystem showed *Rcr1* was associated with the ubiquitin-related proteasome system in plant defence reactions, along with the activation of the calcium-independent MAPK signalling pathway, regulation of ROS production via the activity of protein disulfide isomerases and upregulation of lignin biosynthesis [153].

A high-throughput proteomic study using 2-DGE and MALDI-TOF/TOF MS analyses was performed in the *B. oleracea–P. brassicae* pathosystem to study protein expression during the early stages of host infection, with the highly expressed protein thioredoxin (TRX) enzyme identified, associated with oxidative stress and the pathogen defence response [154]. More than 487 out of 5003 proteins (13.4%) that were identified in *P. brassicae*-infected Chinese cabbage (*B. rapa* var. *pekinensis*) using isobaric tags for relative and absolute quantitation (iTRAQ)-based proteomic analysis were differentially up- or downregulated, and the proteins that contributed to the defence response included those involved in tryptophan and glutathione biosynthesis and cytokinin signalling [155].

In an *H. parasitica* infection study in non-heading Chinese cabbage (*B. campestris* var. *chinensis*), a 2-DGE protein analysis and MALDI-TOF/TOF MS analysis along with transcript mRNA analysis using quantitative RT-PCR suggested a role for a Ca^2+^ signalling pathway as part of the ROS-mediated defence mechanism, with 39% of the genes having no correlation between protein and mRNA levels [156]. Studies have shown that proteomic data may not correlate with transcriptomic data measuring mRNA levels due to post-translational events and protein turnover [155,156]. To determine post-translational protein modification, an online 2D ion-exchange/reversed-phase HPLC method called Multidimensional Protein Identification Technology (MudPIT) can be used [157].

Time-course protein profiling in the pathosystem of *B. juncea*–*Albugo candida* successfully detected proteins that are differentially expressed in the resistant variety such as plant-thaumatin-like protein, superoxide dismutase, glutathione S-transferase, cysteine synthase and red chlorophyll catabolite reductase, suggesting ROS generation plays an important role in *Brassica* host resistance against this pathogen [158].

In a proteomic study applied to a non-pathogenic, arbuscular mycorrhizal fungal species, *Piriformospora indica*, studying the beneficial effect of the fungal endophyte on the *B. napus* host, liquid chromatography-mass spectrometry (LC-MS), coupled with bioinformatics, highlighted significant levels of differentially expressed proteins involved in the stress/defence response during the cell-death colonisation phase in the plant roots, elucidating the role of *P. indica* in enhancing *B. napus* resistance against abiotic and biotic stresses [159]. There is opportunity for the further characterisation of the genes that encode the stress-response proteins expressed in the *B. napus–P. indica* host symbiont relationship. This could be done through the physical mapping of the genes on the *B. napus* genome assemblies supplemented with transcriptome data.

## 3. Application of Omics Technologies in *Brassica* Pathogens

### 3.1. High-Quality Genome Assemblies

High-quality reference genome assemblies for the major pathogens of *Brassica* are currently available, where long-read sequencing approaches such as ONT MinIon sequencing and PacBio sequencing were applied to assemble the genomes of *L. maculans* [160], *S. sclerotiorum* [161], *A. brassicae* [162] and *A. alternata* [163]. The genome of *P. brassicae* was assembled using Illumina Hiseq 2500 technology [164], while the *A. candida* genome was assembled using Roche/454 [165]; both are short-read sequencing technologies. These high-quality genome assemblies of the *Brassica* pathogens revealed that the fungal pathogens contain high genomic variation, including mutations largely induced by transposable elements (TE), large-scale chromosomal re-arrangements [166,167], presence–absence variation [168] and the gain or loss of accessory chromosomes [169,170,171]. These genetic events are continuously and actively evolving in the adaptive response to the selection pressure by the host plant resistance mechanism, thus generating high-genome-plasticity regions, which are often found distributed in compartments where most of the virulence genes are housed [172]. The extent of genetic divergence between individuals of the same fungal plant pathogen species is also high; hence, reference-based mapping is a challenge, although the diverged regions may not always be associated with virulence [173].

The availability of these high-quality genome assemblies has greatly facilitated the discovery of candidate genes for effectors and virulence factors and significantly advanced our understanding about the pathogen in relation to its evolutionary pattern and species diversity through comparative and population genomics studies [137,168,174]. Such deep molecular information will also allow us to uncover the complex *Brassica*–pathogen interactions, as these omics resources are routinely applied in plant pathology research.

### 3.2. Transcriptomics of Virulence-Related Genes

Transcriptome analysis has been increasingly applied to study pathogen gene expression during host invasion, allowing the real-time monitoring of the molecular mechanisms involved in pathogenesis. The recent genome-wide transcriptome analysis of Chittem, et al. [175] highlighted the involvement of peroxisome-related pathways, cell wall degradation by various enzymes and the detoxification of host metabolites as mechanisms of virulence by *S. sclerotiorum* towards *B. napus*. In the *B. napus–L. maculans* interaction, Haddadi, Larkan and Borhan [121] reported the upregulation of genes for fungal toxin biosynthesis during the necrotrophic stage of infection. Furthermore, several *L. maculans* effectors have been predicted from RNA-seq data, consistent with the results of Sonah, et al. [176], who detected different sets of genes coding for several effector proteins, where expression was correlated to *L. maculans* lifestyle transition from a biotrophic to necrotrophic stage. This observation provides an additional clue in deciphering the arsenal of virulence mechanisms employed by *L. maculans*, one of the most notoriously adaptive disease-causing pathogens of the *Brassica* family. In Clubroot, small secreted proteins (SSPbPs) have been identified that were assumed to play critical functions in primary and secondary infections, leading to hypertrophic tissue development [177,178]. For bacterial pathogens, such as *X. campestris* and *P. carotovorum*, a variety of mechanisms are deployed to evade host resistance including the release of extracellular enzymes such as cellulase, mannanase, pectinase, protease, polygalacturonases (PGs) and pectate lyase (Type II secretion system), the injection of effector proteins (Type III secretion system), as well as the production of exopolysaccharides and biofilm formation [179,180,181]. These virulence mechanisms can be manipulated by various techniques such as genome editing or developing cultivars that can undermine such mechanisms.

Through the in silico analysis of comparative genomic and transcriptomic data of *S. sclerotiorum*, 80 putative secondary metabolite gene clusters implicated in virulence in *B. napus* were identified in sub-telomeric regions close to transposable elements, with the upregulation of 12 polyketide synthases (PKSs) and enzymes during *S. sclerotiorum* infection of *B. napus*, revealing clues about the virulence pathway in the *B. napus* host [91]. Enzymes associated with secondary metabolites production in *S. sclerotiorum* to suppress host defence mechanisms, such as PKS, nonribosomal peptide synthase (NRPS) and chalcone synthase (CHS), were upregulated in an RNA-seq experiment studying *B. napus*–*S. sclerotiorum* disease resistance [182].

### 3.3. Secretomics

The fungal plant pathogen secretes a whole suite of proteins, collectively known as the secretome, during its interaction with the host [183]. The secretome comprises effector proteins and specific enzymes crucial for host colonisation, and the composition of each of these secreted proteins may vary between pathogen types based on the pathogen’s mode of nutrition and lifestyle. The identification of these secreted proteins is key to understanding the pathogenicity process of the fungal plant pathogen in the host plant. The availability of rich omics resources and advanced bioinformatics pipelines for diverse fungal plant pathogen species have enabled the quick prediction of effector proteins across kingdom-wide fungal species with different lifestyles and have accelerated the cloning and functional characterisation of candidate effectors [184,185].

An understanding of the structural features of effector proteins, the diversity of the effector genes, and how these genes play a role in the pathogenicity and the evolutionary patterns of the genes are important for uncovering the complexity of the resistance mechanism in *Brassica*–pathogen interactions to support breeding resistant *Brassica* varieties [186]. Due to sequence diversity of effectors for avoiding recognition by the host immune system, the specific function and mechanism of effectors in inducing pathogenicity in the host are difficult to determine. However, the majority of effector genes can be predicted or identified more accurately and effectively based on the known characteristics of cloned effectors using improved computational, bioinformatics software combined with machine learning. Examples include EffectorP 2.0 [187,188], SignalP [189] and ApoplastP [190]. Bioinformatics tools specific for the identification of transposable elements (TEs) have also been developed [191]. Besides proteinaceous effector molecules, non-proteinaceous effectors in fungal pathogens, such as secondary metabolites, small noncoding RNAs and their biological roles in pathogenicity, have also been studied in plant–fungus interactions [192].

The conventional secretion pathway for the proteins during plant–pathogen interaction involves the endoplasmic reticulum–Golgi pathway [193,194]. However, increasing evidence has shown that some secretomes of plant or fungal/oomycete proteins are secreted independently of the classical pathway during plant–pathogen interactions. Hence, it is important that proteins lacking signal peptides within the fungal/oomycete secretome are not overlooked when identifying candidate effectors [195,196].

Some signalling molecules produced by phytopathogenic fungal species that play a part in virulence resemble homologous signalling molecules in the host, acting as mimics to evade the plant immune system for successful disease development [197]. For instance, oxylipins, which are important signalling molecules commonly found in animals, plants and fungi, play a role in growth, development and the defence response, with one of the examples being jasmonate [198]. In *Brassica*, oxylipins were found to display fungicidal activity against *A. brassicae*, *L. maculans*, *S. sclerotiorum* and *Verticillium longisporum* [199]. In phytopathogenic fungi, oxylipins have been found to be involved in disease progression through the modification of the plant host defence mechanisms [200,201], an example being *F. oxysporum* hijacking the oxylipin JA signalling pathway in *A. thaliana* [202].

The gene expression profile from the *P. brassicae* Pb3 genome assembly revealed that the pathogen contains genes that are associated with the biosynthesis of the plant hormones cytokinin and auxin, suggesting a potential role for these hormones in virulence activity in the host plant [203], while gene clusters for the synthesis of the ABA hormone were detected in *L. maculans*, suggesting a putative role of ABA production in disease progression in *B. napus* [204]. One of the well-characterised effectors for *P. brassicae* is the benzoic acid (BA)/SA methyltransferase protein (*PbBSMT*), which suppresses host SA signalling during plant defence [177]. The functional role of *PbBSMT* is similar to that of the SABATH methyltransferase gene family in *A. thaliana*, *AtBSMT1*, where the genes play a role in converting SA into methyl salicylate (MeSA), which is the inactive form of SA, thereby compromising the SAR defence response [205]. A transcriptomic study of *P. brassicae* infection in Kohlrabi (*B. oleracea* var. *gongylodes)* showed that *PbBSMT* was one of the highest-expressed pathogen genes in the root gall tissue, playing a role in the local reduction of SA via *PbBMST*-mediated methylation [206]. It was found from another cloning experiment with *AtBSMT1* vs. *PbBSMT* in *P. brassicae*-infected *Arabidopsis* (host) and *A. candida*-infected *Arabidopsis* (non-host), comparing the level of SA inactivation in *Arabidopsis*, that *PbBSMT* resulted in higher levels of SA inactivation, meaning *PbBSMT* suppressed the host and non-host SAR defence mechanisms at a greater level [206,207]. Multi-omics approaches combining genomics, transcriptomics, proteomics and metabolomics using computational strategies will allow us to identify suitable mimicking molecules in the fungal and/or host species that trigger stronger plant defence systems during plant–pathogen interactions [208].

Beneficial bacterial endophytes found in the apoplast region of *B. napus* have been shown to inhibit the growth of *X. campestris*, *S. sclerotiorum* and *L. maculans*, thus acting as natural biological control against *B. napus* diseases [209]. The high-throughput sequencing of the fungal endophytes obtained from healthy roots of tumorous stem mustard (*B. juncea* var. *tumida*) and *P. brassicae*-infected roots of the same plant species showed a more diverse composition of the fungal endophytes in the healthy roots compared to in the diseased roots. This suggests a strong interaction network in the fungal endophyte community that contributes towards the optimum health of the host plant [210]. A combination of secretomic and proteomic analysis of the apoplast fluids will allow us to identify and characterise the diverse apoplast proteins and further elucidate their role in protecting *B. napus* from pathogen invasion.

### 3.4. Interactome

Interactomics is the study of networks of gene and protein interactions in biological systems [211]. Understanding the biological process and pathogenicity mechanisms of the fungal pathogens in *Brassica* crops is crucial for the identification of disease resistance targets. A web-based database called the Pathogen-Host Interactions database (PHI-base) has been set up that stores curated experimental data obtained from host–pathogen studies, encompassing phenotypic data and biological data on pathogenicity, virulence, and effector gene functions from fungal, oomycete and bacterial pathogens from animal, plant, fungal and insect host species, with embedded search links including BLAST, PubMed, UniProt Knowledgebase and others [212]. Complementing PHI-base, PHI-Nets provides information related to networks of molecular and biological protein–protein interactions for the understanding of pathogenicity and virulence mechanisms in host–pathogen relationships [213].

## 4. Application of Metabolomics and Systems Biology in the *Brassica*–Pathogen System

Metabolomics in plant pathology refers to the study of host plant metabolism changes in response to pathogen infection that provides an understanding of how host–pathogen interaction, through a (de)activation of metabolites and associated signalling pathways, could lead towards a resistant or susceptible outcome for the host [214]. Metabolites associated with black rot infection in *B. oleracea* were identified using liquid chromatography-quadrupole time-of-flight (LC-QTOF)-based metabolite profiling, which revealed that metabolic changes in the host occurred 48 h after infection and implicated photosynthesis, alkaloids, coumarins and sphingolipids as involved during the infection process [215]. Systems biology was constructed to model the metabolic pathway for JA signalling in the *Brassica*–*Alternaria* pathophysiology to identify important elements in the regulation of resistance mechanisms and to pinpoint molecular targets for engineering enhanced resistance in *Brassica* crops [216].

**Figure 2:**
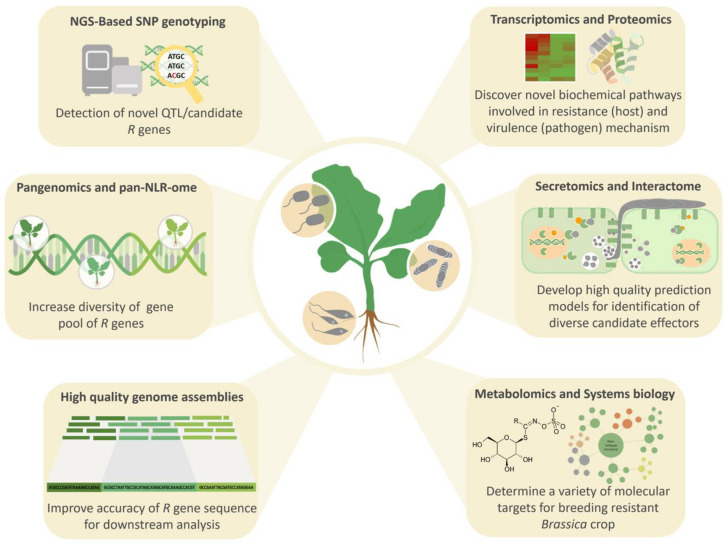
The application of multi-omics technologies in the discovery of novel plant–pathogen interactions in the *Brassica* pathosystems.

The huge number of data collected from multi-omics technologies will be useful in the construction of network biology, where mathematical models and computational approaches are implemented to predict the pathogenicity and virulence mechanisms in plant–pathogen interactions [217]. Metabolic pathways supported by mathematical modelling can be used to study how cells within a multicellular organism work cooperatively to carry out a particular function. An example of systems biology was carried out for a *A. thaliana*–*S. sclerotiorum* pathosystem, where a genome-scale metabolic model of *S. sclerotiorum* based on global gene expression was constructed to assess the metabolic activity in different parts of the hyphal cells, supporting the hypothesis that cooperation in *S. sclerotiorum* hyphal cells is necessary for virulence and host colonisation [218]. A combination of metabolomics with quantitative genetics was used to discover the potential role of gluconasturtiin in the *B. napus* resistance response against Clubroot and the underlying QTL controlling the trait on chromosome C03 and C09 [219]. Gluconasturtiin is a type of glucosinolate compound associated with the biotic resistance responses of *Brassica* species [220]. An example of using multi-omics supplemented with functional studies to discover key resistance pathways involved the soybean–*S. sclerotiorum* pathosystem, where the induction of JA signalling, elevated ROS control and reprogramming of the phenylpropanoid pathway have been suggested to be an important resistance mechanism [221]. Many more novel plant–pathogen interactions in the *Brassica* pathosystems could be discovered through the application of multi-omics technologies (Figure 2).

## 5. Future Perspectives

Genomics-assisted breeding, also known as genomic selection, is an advanced level of omics breeding in *Brassica* crops. Genomic selection incorporating multiple traits in crop breeding programs, with a focus on biotic stress, not only offers a promising strategy for developing high-quality *Brassica* crops resilient against a wide variety of pathogen types, but does so without compromising yield or crop quality [222,223]. A further area of interest is to screen for favourable alleles of diverse resistance genes sourced from wild relatives of *Brassica* species or, beyond that, wider members of the Brassicaceae family and expand from disease resistance genes to regulators such as small RNAs to find out how the disease resistance gene expression is being regulated [224].

Beyond the gene level, a wider perspective on how epigenetics affects plant responses towards pathogen attack should be considered to enhance our understanding about the resistance mechanisms in *Brassica*. Examples include how TEs, which are associated with DNA methylation, might contribute towards the resistance and susceptibility of the *Brassica* crop [225,226] and how epigenetic variability is linked to phenotypic responses towards plant pathogens, as shown in the *Arabidopsis*–*P. brassicae* pathosystem, where DNA methylation contributes towards quantitative resistance to Clubroot, based on epigenotyped epigenetic recombinant inbred lines [227]. In the *B. napus*–*L. maculans* pathosystem, promoters of defence genes were differentially methylated during the early stages of infection in the resistant host cultivar compared to the susceptible cultivar [228]. From the pathogen perspective, the availability of the multi-omics data is useful for screening candidate pathogenicity genes in the pathogen. However, this is dependent upon the race classification keeping pace with the omics data of the fungal/oomycete pathogens becoming available; otherwise, an effective application of the pathogenicity genes in field populations cannot be achieved [229].

Functional analysis of candidate genes in the *Brassica*–pathogen system can be performed using the clustered, regularly interspaced, palindromic repeats (CRISPR)-Cas9 (CRISPR-associated protein 9) system. In *B. napus*, two genes, *BnWRKY11* and *BnWRKY70*, that encode for WRKY transcription factors were successfully knocked out using CRISPR-Cas9, showing the latter gene regulates disease resistance to *S. sclerotiorum* [230]. This multiplex gene knock-out study is very useful for gene functional studies in polyploid crops such as *Brassica* that contain many copies of the same genes as homeologs within the genome. The CRISPR-Cas9 system was also successfully applied in *B. rapa* for early-flowering trait genome editing [231]. In *L. maculans*, the chitin-binding gene *LmCBP1*, which was highly expressed during infection in *B. napus* and functional analysis using CRISPR-Cas9, was shown to play a role in enhancing cell death for pathogen growth and also to be involved in tolerance towards H_2_O_2_ during the plant immune response [232].

## 6. Conclusions

The omics tools have advanced our understanding of *Brassica*–pathogen interactions in many ways and will become the mainstream approach to rapidly identifying QTL/candidate *R*/pathogenicity genes for breeding superior *Brassica* crop species that are resistant towards the main pathogen types discussed in this review. Using the various omics or multi-omics tools, supplemented with further fine tuning of the bioinformatics methods, will not only speed up the screening of favourable alleles in *Brassica* germplasm promoting resistance against the major pathogens, but also expediate the identification and cloning of favourable genes with increased precision. Together, this will foster success in the breeding of improved and/or new varieties of *Brassica* crops for sustainable agriculture [233].

## Figures and Tables

**Table 2:** Summary of application of next-generation sequencing (NGS)-based SNP genotyping approaches in resistance studies of *Brassica* diseases.

Approach	Disease Type	*Brassica* Sample	Main Findings	Reference
WGRS, SNP genotyping	-	991 *B. napus* worldwide accessions	Selective-sweep regions enriched with genes related to stress response	Wu, et al. [82]
WGRS, SNP genotyping	-	588 *B. napus* worldwide accessions	A sub-genomic-specific selection contributes towards biotic stress response with several candidate genes identified	Lu, et al. [83]
WGRS, QTL mapping	Black rot	Mapping population of cabbage *B. oleracea* var. *capitata* inbred lines “C1234” (resistant) and “C1184” (susceptible)	21 candidate NBS-LRR genes associated with black rot resistance in *B. oleracea*	Lee, et al. [84]
GBS, GWAS	Blackleg	243 *B. napus* accessions from Canada and China	Significant SNPs were found on chromosome A08 with 25 RGAs identified consisting of NBS, RLK, RLP and TM-CC type *R* genes	Fu, et al. [73]
GBS, GWAS	Sclerotinia	*B. juncea–B. fruticulosa* introgression lines	20 candidate genes mostly located on the A sub-genome of *B. juncea*	Atri, et al. [75]
GBS, GWAS	Sclerotinia	*B. juncea–Erucastrum cardaminoides* introgression lines	QTL region on chromosomes A03 and B03 and candidate genes being LRR-RLK, LRR-PK and TIR-NBR-LRR	Rana, et al. [76]
tGBS^®^, GWAS	-	135 *B. oleracea* accessions including var. broccoli, Brussels sprout, cabbage, cauliflower, Chinese kala, kale, kohlrabi and savoy cabbage	Resistant phenotype mostly found in kale. Candidate genes encoding pathogenesis-related proteins were mainly found on chromosome C07	Farid, et al. [85]
Brassica 60K SNP array, GWAS	Clubroot	Mapping population of cabbage *B. oleracea* inbred lines “263” and “GZ87”	Significant QTL and novel loci found on C sub-genome	Peng, et al. [78]
Brassica 60K SNP array, linkage disequilibrium (LD) analysis	-	327 *B. napus* worldwide accessions comprising three ecotypes	Selective-sweep regions enriched with Blackleg and Sclerotinia resistance QTLs	Wei, et al. [86]
Brassica 60K SNP array, GWAS	Clubroot	472 *B. napus* worldwide accessions	Most candidate genes were found on C sub-genome with novel QTLs and TIR-NBS gene clusters	Li, et al. [77]
Brassica 60K SNP array, GWAS	Sclerotinia	448 worldwide *B. napus* accessions	Two novel loci with 39 candidate genes on C sub-genome	Wu, et al. [74]
Brassica 60K SNP array	Blackleg	Seven *B. napus* seven donor parents for introgression lines	Genomic background of individual varieties and multiple defence-related gene interactions influence the resistance levels	Larkan, et al. [87]
